# SS‐31 and NMN: Two paths to improve metabolism and function in aged hearts

**DOI:** 10.1111/acel.13213

**Published:** 2020-08-11

**Authors:** Jeremy A. Whitson, Alessandro Bitto, Huiliang Zhang, Mariya T. Sweetwyne, Rene Coig, Saakshi Bhayana, Eric G. Shankland, Lu Wang, Theo K. Bammler, Kathryn F. Mills, Shin‐Ichiro Imai, Kevin E. Conley, David J. Marcinek, Peter S. Rabinovitch

**Affiliations:** ^1^ Department of Laboratory Medicine and Pathology University of Washington Seattle Washington USA; ^2^ Department of Radiology University of Washington Seattle Washington USA; ^3^ Department of Environmental & Occupational Health Sciences University of Washington Seattle Washington USA; ^4^ Department of Developmental Biology Washington University School of Medicine St. Louis Missouri USA

**Keywords:** aging, heart, magnetic resonance spectroscopy, metabolomics, NMN, SS‐31

## Abstract

The effects of two different mitochondrial‐targeted drugs, SS‐31 and NMN, were tested on Old mouse hearts. After treatment with the drugs, individually or Combined, heart function was examined by echocardiography. SS‐31 partially reversed an age‐related decline in diastolic function while NMN fully reversed an age‐related deficiency in systolic function at a higher workload. Metabolomic analysis revealed that both NMN and the Combined treatment increased nicotinamide and 1‐methylnicotinamide levels, indicating greater NAD^+^ turnover, but only the Combined treatment resulted in significantly greater steady‐state NAD(H) levels. A novel magnetic resonance spectroscopy approach was used to assess how metabolite levels responded to changing cardiac workload. PCr/ATP decreased in response to increased workload in Old Control, but not Young, hearts, indicating an age‐related decline in energetic capacity. Both drugs were able to normalize the PCr/ATP dynamics. SS‐31 and NMN treatment also increased mitochondrial NAD(P)H production under the higher workload, while only NMN increased NAD^+^ in response to increased work. These measures did not shift in hearts given the Combined treatment, which may be owed to the enhanced NAD(H) levels in the resting state after this treatment. Overall, these results indicate that both drugs are effective at restoring different aspects of mitochondrial and heart health and that combining them results in a synergistic effect that rejuvenates Old hearts and best recapitulates the Young state.

## Graphical abstract

The effects of the mitochondrial‐targeted drugs SS‐31 and NMN were tested on aged mouse hearts. It was found that SS‐31 restores diastolic function, while NMN restores high work systolic function. Both drugs normalized PCr/ATP dynamics and increased mitochondrial NAD(P)H levels in response to a work jump. NMN also raised NAD^+^ levels during the work jump. When given in combination, the drugs increased resting NAD(H) levels, indicating a synergistic effect.

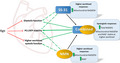

## 1 INTRODUCTION

Cardiovascular disease remains the major cause of mortality in older adults (Heron & Anderson, 2016; Tinetti et al., 2012), eclipsing all forms of cancer by the age of 75 (Roger et al., 2011). Furthermore, age‐related decline in heart function, which includes dysfunction in both diastole and systole (Dai, Chen, Johnson, Szeto, & Rabinovitch, 2012), has been shown to be strongly associated with frailty (Leibowitz et al., 2016). Because of the immense burden of heart dysfunction on the elderly population, the development of treatments that can improve heart health in aged individuals remain a top priority for extending human healthspan.

The majority of the heart's volume consists of cardiomyocytes (Zhou & Pu, 2016), which must meet the incredible energetic demand of continuous contraction and relaxation throughout an entire lifespan. To meet this energetic need, heart tissue has one of the highest mitochondrial densities found in the body (Benard et al., 2006). As cells age, nicotinamide dinucleotide (NAD^+^) levels, ATP output, and mitochondrial biogenesis decrease, while oxidative stress, mtDNA damage, and mitochondrial structural alterations increase (Chistiakov, Sobenin, Revin, Orekhov, & Bobryshev, 2014). Based on these physiological features, it has long been hypothesized that mitochondrial fitness is key to maintaining heart health (Herrmann & Decherd, 1939). According to this hypothesis, treatments that can restore mitochondrial parameters to more youthful states in aged cardiomyocytes have the potential to repair the function of aged hearts.

In this study, we set out to determine the functional and metabolic effects on aged mouse hearts of two mitochondrial‐targeted drugs proposed to treat age‐related dysfunction: SS‐31 and nicotinamide mononucleotide (NMN).

SS‐31, also known as elamipretide, is a synthetic tetrapeptide consisting of D‐arginine, 2′,6′‐dimethyl‐l‐tyrosine, lysine, and phenylalanine that associates with the head group of cardiolipin, an essential phospholipid specific to the mitochondrial inner membrane (Szeto, 2014). Previous testing of SS‐31 in mammals has shown that the drug decreases oxidative stress (Hou et al., 2016) and protects against ischemia–reperfusion injury (Cai et al., 2018), although the specific mechanisms by which the drug elicits these changes are still being actively investigated. There are indications that a combination of preventing cytochrome c peroxidase activity, promoting cristae, and electron transport chain complex organization, as well as influencing other mitochondrial proteins, contributes to these beneficial effects (Birk, Chao, Bracken, Warren, & Szeto, 2014; Szeto, 2014). We have recently demonstrated that SS‐31 improves diastolic function and reduces oxidative stress in the hearts of aged mice when delivered for 8 weeks (Chiao et al., 2020).

NMN is a naturally occurring nucleotide precursor of NAD^+^ that is generated via the NAD^+^ salvage pathway (Yoshino, Baur, & Imai, 2018). NMN and its precursor nicotinamide riboside (NR) have been hypothesized to improve mitochondrial health by increasing cellular NAD^+^ availability and therefore improving energetic capacity and activating sirtuin deacetylase activity. Recent studies have provided strong evidence for this hypothesis and shown that NMN supplementation bolsters NAD^+^ availability and improves many classical signs of aging in mice (Mills et al., 2016). NMN supplementation has also been shown to partially restore heart function in a mouse model of heart failure (Lee et al., 2016).

Although NMN and SS‐31 both target mitochondria, they do so via different mechanisms of action. Thus, we hypothesized that the drugs would differ in effect on the heart and may have a synergistic effect when applied together. We sought to compare and contrast the mechanism and effects of these drugs in order to better understand each individually and how age‐related deficits in heart function can be addressed.

Because mice recapitulate many aspects of human aging (Vanhooren & Libert, 2013), including a general decline in heart function (Dai et al., 2012), they are an excellent model for determining whether these drugs have the potential to rejuvenate aged hearts in humans and to determine the mechanisms of this improvement. To test the effects of these drugs on the aged heart, mice aged 24 months at the start of treatment (Old) were randomly sorted into Control, SS‐31, NMN, or Combined (SS‐31 + NMN) treatment groups for comparison with mice 5–6 months of age (Young). Treatment doses of SS‐31 and NMN were in alignment with those that have previously been shown to be effective in other healthspan measures (Chiao et al., 2020; Mills et al., 2016). In vivo magnetic resonance spectroscopy is typically used to examine ATP and PCr levels in muscle tissue, though when applied to beating hearts the spectra must be voxel‐localized and timed to coincide with heart and pulmonary cycles. In this report, we further developed and applied for the first time an MRS technique to determine how additional heart metabolites, including nicotinamide nucleotides, respond to an increase in cardiac workload, defined by an accelerated heart rate, in vivo. This analysis was coupled with measurements by echocardiography and liquid chromatography tandem mass spectrometry (LC‐MS/MS) to obtain an integrated view of changes resulting from age and drug treatment from the metabolic to the functional level in the heart.

## 2 RESULTS

### 2.1 SS‐31 and NMN treatments result in different functional improvements

**FIGURE 1:**
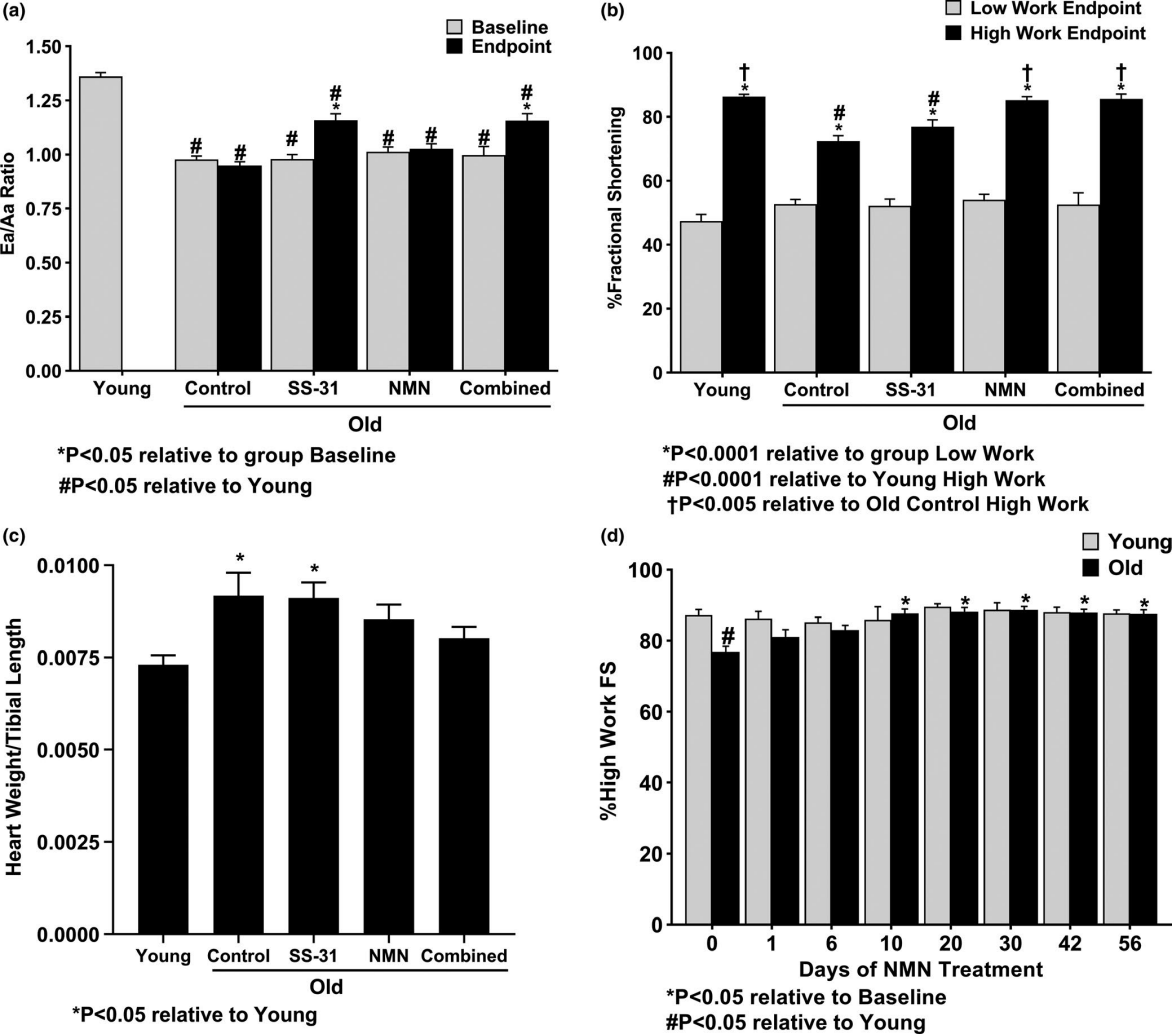
Effect of SS‐31 and NMN on heart function. (a) Ea/Aa ratio at baseline (treatment day 0) and endpoint (8 weeks of treatment) and (b) percent fractional shortening at low work (‐dobutamine) and high work (+dobutamine) at the 8 week treatment endpoint, measured by echocardiography. *N* = 29 Young, 49 Old Control, 29 Old SS‐31, 28 Old NMN, 10 Old Combined. (c) High work (+dobutamine) percent fractional shortening measured by echocardiography in Young and Old mice at 0, 1, 6, 10, 20, 30, 42, and 56 days of NMN treatment. *N* = 8 Young, 19 Old. (d) Ratio of heart weight (g) to tibia length (mm) assessed at endpoint (8 weeks of treatment) as a measure of cardiac hypertrophy. *N* = 21 Young, 21 Old Control, 16 Old SS‐31, 15 Old NMN, 14 Old Combined.

Echocardiography was performed on all mice at baseline and again on Old mice after 8 weeks of treatment. Diastolic function was assessed by determining the ratio of blood flow across the mitral valve in early diastole (Ea) to the flow in late diastole (Aa). Ea/Aa is one of the primary markers used when assessing diastolic function in human patients (Mitter, Shah, & Thomas, 2017) and is the parameter of diastolic function that has been used in composing a comprehensive frailty index of mouse aging (Kane, Keller, Heinze‐Milne, Grandy, & Howlett, 2019). Old Control mice had a significantly (*p* < 0.05) lower Ea/Aa compared to Young mice at their baseline, indicating a decline in diastolic function with age. As found previously (Chiao et al., 2020), Old mice treated with SS‐31 showed a significant (*p* < 0.05) improvement in Ea/Aa, restoring it approximately halfway to that of Young Ea/Aa values, whereas the Control and NMN‐treated groups showed no change in this parameter (Figure 1a). The Combined treatment group recapitulated the Ea/Aa change resulting from SS‐31 alone but did not show any further improvement.

Systolic function was assessed by measuring percent fractional shortening (i.e., the degree to which the ventricular lumen closes during systole) from echocardiograms. Since anesthetized mice have subphysiological heart rates, alterations in systolic function may not be manifested under these low work conditions. Thus, fractional shortening was also measured following injection of the ionotropic agent dobutamine at a concentration that produced higher heart rates within the normal physiological range (Figure 1b). Long‐term assessment of heart rates showed that dobutamine administration results in a similar significant (*p* < 0.001) increase in heart rate across all groups (Figure S1). There were no significant differences in fractional shortening between the groups at the low work state or within groups at treatment endpoints relative to their baselines (Figure S2). Under higher workload conditions, however, Old Control mice manifested a significantly (*p* < 0.0001) lower percent fractional shortening than Young mice, indicating a decline in high work systolic function with age. SS‐31 treatment did not significantly affect fractional shortening, but NMN and the Combined treatments both significantly (*p* < 0.005) improved high work percent fractional shortening in Old mice, fully restoring it to Young levels (Figure 1b).

While our previous studies have shown that SS‐31 induces a partial effect on diastolic function after 4 weeks of treatment and appears to reach its peak effect at 8 weeks (Chiao et al., 2020), it was unknown on what timescale the cardiac functional improvements induced by NMN occur. To test this, Young and Old mice were treated with NMN and tested at regular intervals to determine changes in their high work percent fractional shortening (Figure 1c). After 10 days of treatment, Old mice treated with NMN showed a significant (*p* < 0.05) improvement in high work fractional shortening, restoring it to Young levels. This remained constant, with no further enhancement from prolonged treatment. Young fractional shortening was unaffected by NMN treatment. Young mice were also given SS‐31 to determine whether there was any effect on diastolic function, but the Ea/Aa ratio was not significantly changed after 4 or 8 weeks of SS‐31 treatment (Figure S3). The persistence of the functional improvement induced by NMN treatment was also examined. In contrast to SS‐31, which we have previously shown to maintain half its functional effect 4 weeks after cessation of treatment (Chiao et al., 2020), NMN's effect on aged hearts is more transient. After stopping NMN treatment, high work fractional shortening remained the same one day later but then declined at approximately the rate it had improved after starting treatment, matching the baseline for Old hearts after 10 days without treatment (Figure S4).

Relative to Young mice, Old Control mice showed a significant (*p* < 0.05) increase in cardiac hypertrophy, measured as the ratio of heart weight to tibia length (Figure 1d). NMN and the Combined treatments appeared to reduce this hypertrophy and partially restore heart weight/tibia length to the Young state, but SS‐31 alone did not have a significant effect.

Mitochondrial function in isolated cardiomyocytes from Old Control and NMN‐treated hearts was assessed by Seahorse assay (Agilent Technologies, Santa Clara, CA). While we have previously shown by this method that SS‐31 normalized age‐related increases in basal respiration and proton leak (Chiao et al., 2020), NMN treatment did not appear to have any such effect, as mitochondrial oxygen consumption appeared unchanged from the Control group by all measures (Figure S5).

### 2.2 Metabolomic analyses of hearts reveal unique changes resulting from SS‐31 and NMN treatments

Analysis of cardiac metabolic differences at the endpoint of treatment was completed by two LC‐MS/MS methods: (a) a focused analysis of NAD‐related metabolites and (b) a general screening of 369 common metabolites.

**FIGURE 2:**
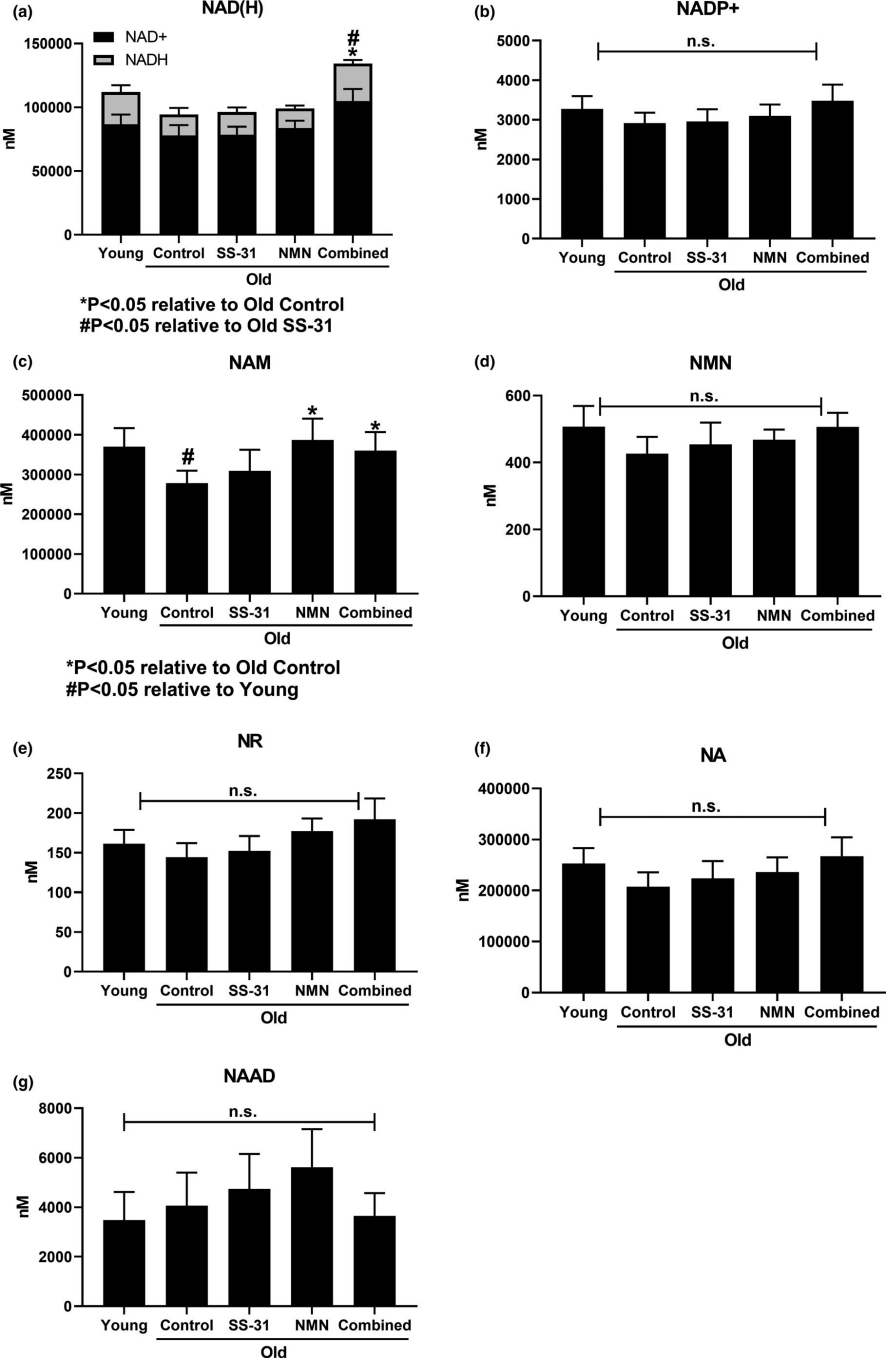
Analysis of endpoint NAD^+^‐related metabolite levels. Endpoint (8 weeks of treatment) levels of (a) NAD(H), (b) NADP^+^, (c) NAM, (d) NMN, (e) NR, (f) NA, and (g) NAAD in mouse hearts as measured by targeted LC‐MS/MS. The height of the stacked columns in (a) indicates the total NAD+NADH content. The significance noted is based on these total values. *N* = 11 Young, 10 Old Control, 11 Old SS‐31, 11 Old NMN, 8 Old Combined.

By the first method, the total NAD(H) pool of the heart appeared to decline with age (Figure 2a), as has been reported elsewhere (Braidy et al., 2011). Neither SS‐31 nor NMN treatment alone resulted in any change to total NAD(H). However, when NMN and SS‐31 were given in combination, there was a significant (*p* < 0.05) increase in NAD(H) levels. The ratio of NAD^+^ to NADH did not change significantly in any of the groups. NADP^+^ also did not show any significant changes between the groups (Figure 2b), and NADPH was undetectable by this assay. Of the assayed NAD precursors, only nicotinamide (NAM) showed a difference between groups, with a significant (*p* < 0.02) decrease in levels between Young and Old Control hearts, and a restoration by both NMN and Combined treatments (*p* < 0.05) (Figure 2c). NMN, nicotinamide riboside (NR), and nicotinic acid (NA) also showed the same trend of a decrease with age and restoration by NMN and Combined treatments but did not individually reach significance (Figure 2d–f). Nicotinamide adenine dinucleotide (NAAD) did not follow this trend and did not show any significant changes (Figure 2g).

**FIGURE 3:**
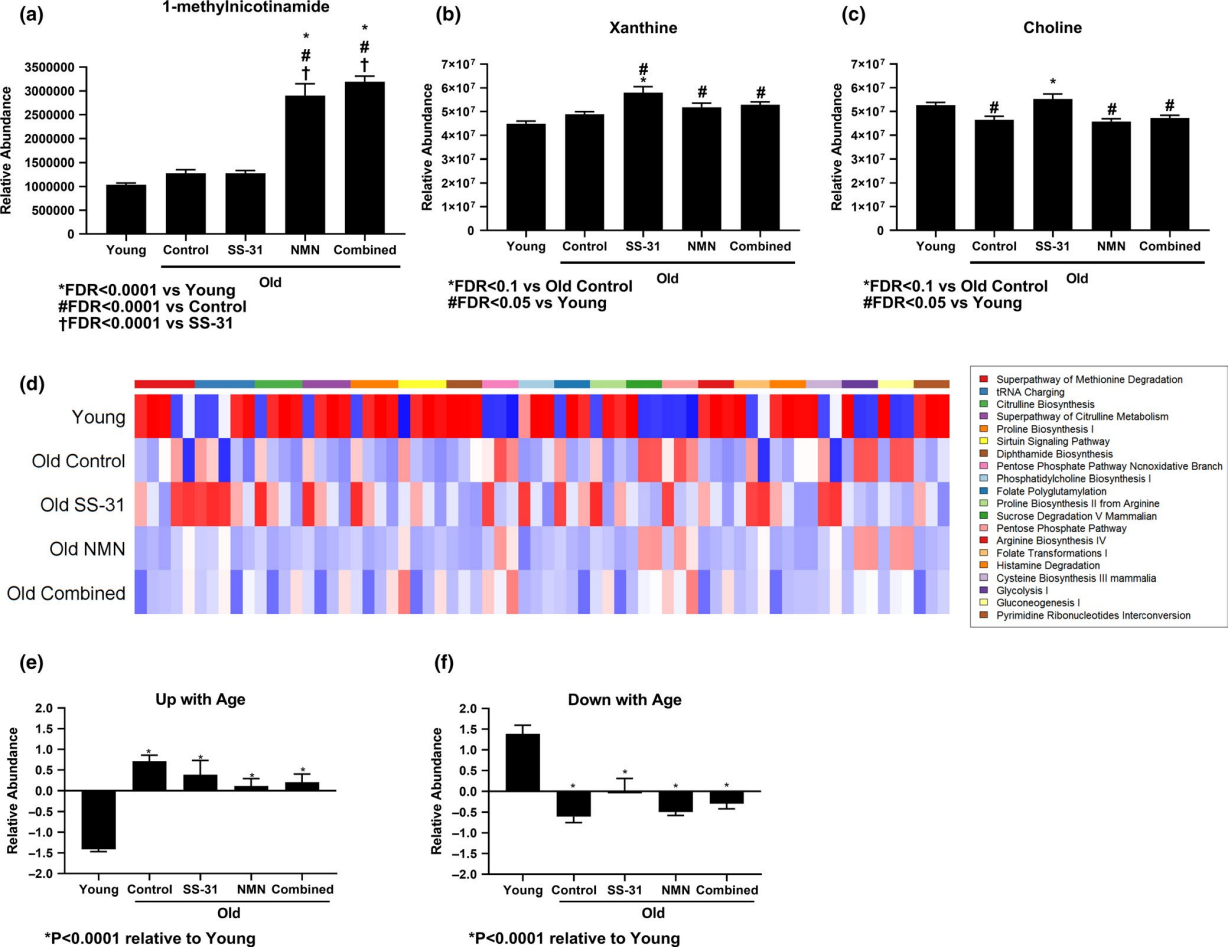
General metabolomics analysis of mouse hearts. Endpoint (8 weeks of treatment) levels of (a) 1‐methylnicotinamide (MNA), (b) xanthine, and (c) choline as measured by general targeted metabolomics. *N* = 12 Young, 10 Old Control, 12 Old SS‐31, 11 Old NMN, 8 Old Combined. Means are calculated from postprocessed values and are not log2‐transformed. (d) Heatmap of metabolic changes in the top 20 canonical pathways based on all changes with an unadjusted *p* < 0.05 when comparing Young to Old Control. (e and f) Quantification of the heatmap, separated into metabolites that decreased (e) or increased (f) with age.

In the general targeted metabolomics assay, which primarily assessed metabolites involved in protein and carbohydrate energy metabolism, 26 metabolites showed significant (FDR < 0.1) changes when comparing Young to Old Control mice (Table 1). None of the treatments appeared to have a significant effect on shifting the overall metabolome of Old hearts back toward the Young state, and each showed only a few significant (FDR < 0.1) changes. Most notably, the NMN and Combined treatments resulted in a sharp increase in 1‐methylnicotinamide (MNA) (FDR < 0.0001) in Old mice compared to Young, Old Control, and Old SS‐31 mice (Figure 3a). SS‐31 treatment resulted in modest increases in xanthine and choline (FDR < 0.1) levels (Figure 3b,c). Intriguingly, the Combined treatment did not recapitulate the SS‐31‐induced changes and instead showed similar levels of both choline and xanthine to the Old Control and Old NMN groups. The full set of comparisons between all groups can be found in the Appendix S1. We investigated whether the change in MNA levels could be due to a difference in nicotinamide N‐methyltransferase (NNMT) levels; however, this enzyme was not detectable in any mouse hearts by Western blot (Figure S6) or shotgun proteomics (data not shown).

**TABLE 1:** Significant age‐related metabolomics changes in mouse hearts

Metabolite	Fold change relative to old control			
	Young	Old SS−31	Old NMN	Old combined
5‐Aminovaleric acid	**2.09(0.38)**	1.79(0.38)	1.76(0.38)	1.81(0.41)
Carnosine	**2.00(0.15)**	0.78(0.16)	0.84(0.16)	0.94(0.17)
N‐Formylmethionine	**1.93(0.32)**	1.60(0.32)	1.62(0.33)	1.79(0.35)
Hypotaurine	**1.92(0.21)**	1.16(0.22)	1.23(0.22)	1.35(0.23)
Beta‐alanine	**1.78(0.15)**	0.95(0.15)	1.21(0.15)	1.12(0.16)
Anserine	**1.64(0.10)**	0.80(0.11)	0.89(0.11)	0.82(0.11)
Adenine	**1.56(0.16)**	1.09(0.17)	1.03(0.17)	1.21(0.18)
Cholesterol sulfate	**1.46(0.19)**	1.14(0.19)	1.08(0.19)	1.11(0.20)
Ethylmalonic acid	**1.39(0.18)**	1.00(0.18)	1.29(0.18)	1.06(0.19)
5′‐Methylthioadenosine	**1.35(0.14)**	1.00(0.14)	0.96(0.14)	1.05(0.15)
ADP	**1.32(0.08)**	1.07(0.08)	1.02(0.08)	1.02(0.09)
Citrulline	**1.29(0.10)**	1.01(0.10)	1.04(0.10)	1.13(0.11)
L‐Glutamic acid	**1.20(0.10)**	1.11(0.10)	0.98(0.10)	0.94(0.11)
Choline	**1.14(0.06)**	**1.19(0.06)**	0.99(0.06)	1.02(0.07)
L‐Serine	**0.81(0.10)**	1.06(0.10)	0.90(0.10)	0.89(0.11)
Sedoheptulose 7‐phosphate	**0.77(0.13)**	1.20(0.13)	0.93(0.13)	1.04(0.14)
3‐Methylhistidine	**0.65(0.16)**	0.89(0.16)	0.97(0.16)	0.87(0.18)
Pipecolic acid	**0.63(0.20)**	0.97(0.20)	0.86(0.20)	0.90(0.21)
Dihydroxyacetone phosphate	**0.59(0.14)**	0.72(0.14)	0.93(0.14)	0.81(0.16)
Lactose/trehalose	**0.55(0.25)**	0.81(0.26)	0.99(0.26)	1.14(0.28)
Glyceraldehyde‐3‐phosphate	**0.54(0.16)**	0.71(0.16)	0.91(0.16)	0.78(0.17)
Erythrose 4‐phosphate	**0.50(0.28)**	0.75(0.29)	0.91(0.29)	1.03(0.31)
Glucose‐6‐phosphate	**0.49(0.29)**	0.77(0.29)	0.93(0.29)	1.05(0.31)
Trimethyloxamine	**0.47(0.31)**	1.33(0.31)	1.14(0.31)	1.07(0.34)
Glucose‐1,6‐bisphosphate	**0.40(0.32)**	0.56(0.32)	0.90(0.32)	0.61(0.34)
L‐Cystine	**0.32(0.43)**	0.85(0.43)	0.58(0.44)	0.62(0.47)

Values are untransformed fold changes with standard errors in parentheses. Bold numbers indicate significance (FDR < 0.1). Ordered by fold change. Full results can be found in the Appendix S1.

Analysis of general trends in canonical pathways from this dataset was performed based on all changes *p* < 0.05 (unadjusted *p*‐values) in Young relative to Old Control hearts using Ingenuity Pathway Analysis (Qiagen, Hilden, Germany). This revealed that the major metabolic pathways being affected by age included glycolysis, citrulline biosynthesis, phosphatidylcholine biosynthesis, and others (Figure 3d). Quantification of relative changes in the canonical pathways heatmap was split into metabolites that increased with age (Figure 3e) and those that decreased with age (Figure 3f). While there were suggestive trends toward reversal of the age‐related changes to these pathways, treatment effects did not reach significance in the quantification. Mean difference plots for each comparison are provided in the supplemental data (Figure S7).

### 2.3 Magnetic resonance spectroscopy probes dynamic metabolic shifts in vivo

**FIGURE 4:**
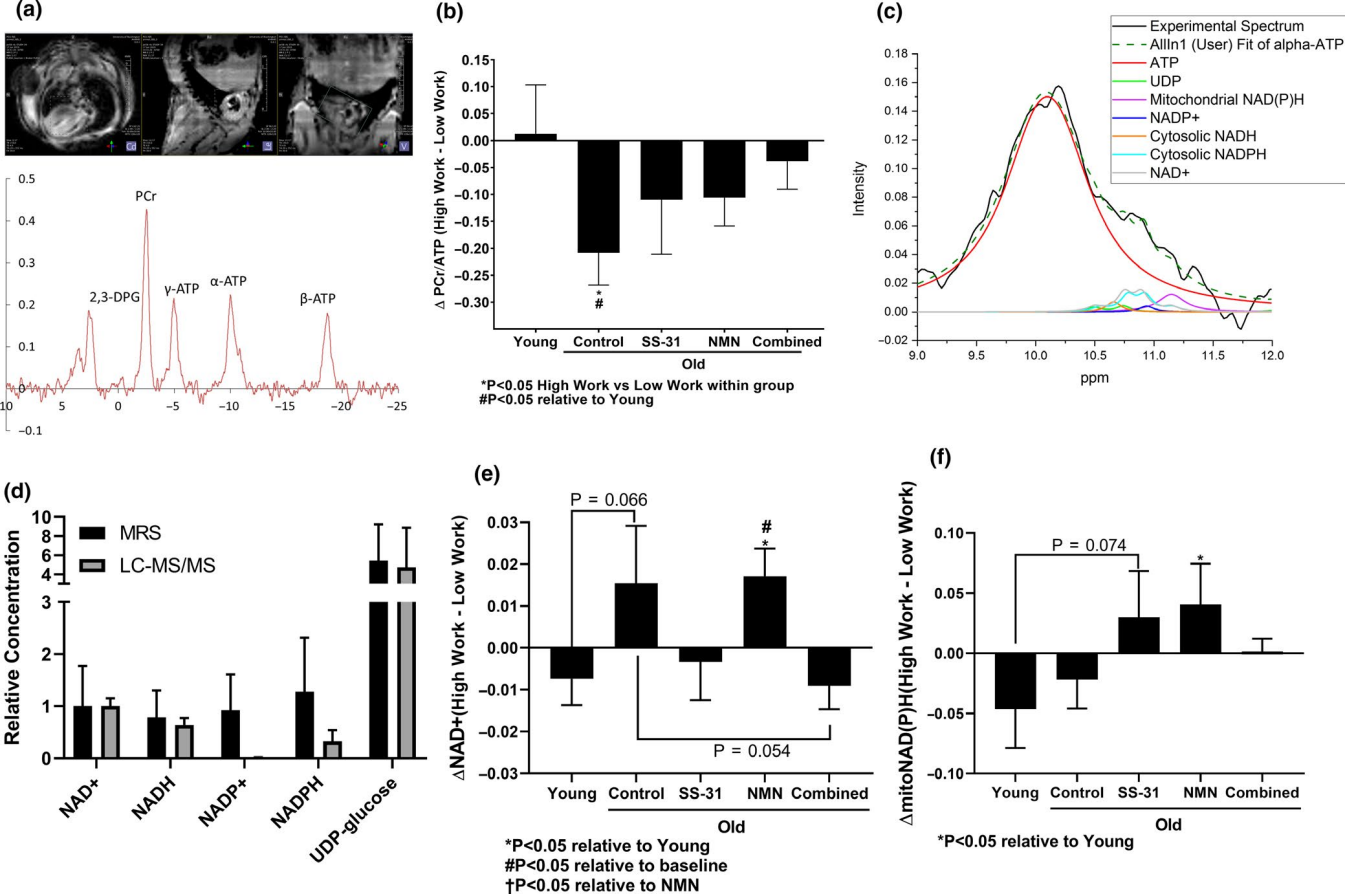
Magnetic resonance measurements of dynamic mitochondrial metabolism. (a) Representative ^1^H‐magnetic resonance imaging of mouse chest cavity, heart voxel localization for ISIS, and resulting ^31^P spectrum. (b) Change in PCr/ATP ratio after injection of dobutamine. *N* = 11 Young, 11 Old Control, 11 Old SS‐31, 13 Old NMN, 10 Old Combined. (c) Representative image of the automated fitting model. (d) Comparison of MRS and metabolomics Old Control results. *N* = 11 MRS, 10 metabolomics. (e and f) Change in (e) NAD^+^ and (f) mitochondrial NAD(P)H after injection of dobutamine. *N* = 11 Young, 10 Old Control, 9 Old SS‐31, 11 Old NMN, 10 Old Combined.

^31^P**‐**magnetic resonance spectroscopy (MRS) was applied at the treatment endpoint in order to probe how metabolites in the heart respond to a dobutamine‐induced increase in cardiac workload in vivo. Image‐Selected In Vivo Spectroscopy (ISIS) was used to collect data localized to the heart with minimal inclusion of surrounding tissues. A representative heart‐localized voxel and resulting MRS spectrum from this technique are shown in Figure 4a.

The principle difference in the energetic response to the increased heart rate was measured as the ratio of the peak areas of phosphocreatine (PCr) to γ‐ATP (given as PCr/ATP; Figure 4b). This measurement provides an assessment of how effectively the heart can respond to increased work by determining the degree to which the high‐energy phosphates stored in PCr are depleted from cardiomyocytes under increased demand for ATP. While Young mouse hearts showed no change in PCr/ATP in response to increased workload, Old Control mouse hearts had a significant (*p* < 0.05) decrease in PCr/ATP following dobutamine injection, indicating a diminished ability of the heart to handle such a workload with age. Although still showing a slight decrease in PCr/ATP at the higher workload, the SS‐31, NMN, and Combined treatments all appeared to mitigate the age‐related change, and PCr/ATP in the treated groups was not significantly different from the Young state or their predobutamine baselines.

In order to analyze how less abundant metabolites in the heart respond to increased cardiac workload in these mice, peaks corresponding to chemical standards of α‐ATP, UDP‐glucose, NAD^+^, NADH, NADP^+^, and NADPH were fit to the experimental spectra for quantification using a custom model in OriginPro software (OriginLab, Northampton, MA). An additional peak corresponding to mitochondrial NAD(P)H was also fit to the data, based upon our prior study demonstrating the presence of this peak in vivo, which has an altered chemical shift relative to cytosolic NAD(P)H due to the unique environment of the mitochondrial matrix (Conley, Ali, Flores, Jubrias, & Shankland, 2016). A representative fit of experimental data using this model is shown in Figure 4c. To assess the performance of this technique for analyzing these metabolites, MRS results from Old Control mice prior to dobutamine injection were compared with those from the general metabolomics screen, which also analyzed NAD(P)(H) and UDP‐glucose (Figure 4d). Relative levels (normalized to mean NAD^+^ for comparison across methods) of NAD^+^, NADH, and UDP‐glucose determined by both techniques were remarkably similar, but there was a large discrepancy in NADP^+^ and NADPH levels, which were much higher relatively when measured by MRS. This is unsurprising given the known issues with extracting and preserving these metabolites from tissue samples (Lu, Wang, Chen, Hui, & Rabinowitz, 2018) and thus may actually demonstrate a strength of the in vivo MRS method. Mitochondrial NAD(P)H cannot be distinguished from the cytosol‐localized molecules by the metabolomics method and thus was not included in the comparison. Overall, this comparison supports the relative quantification of these metabolites from in vivo heart MRS spectra.

The MRS data showed a trend of NAD^+^ remaining stable or decreasing slightly in Young hearts with higher workload but increasing in Old Control hearts (*p* = 0.066) (Figure 4e). SS‐31 and the Combined treatment appeared to reverse this age‐related change in NAD^+^ dynamics. Conversely, NMN treatment did not reverse the age‐related change and the increase in NAD^+^ at higher workload in these mice was significantly (*p* < 0.05) different from the Young state at higher workload. Mitochondrial NAD(P)H also showed changing dynamics with drug treatment (Figure 4f). While Young and Old Control hearts had a trend of slightly decreased mitochondrial NAD(P)H with the increased heart rate, SS‐31 and NMN‐treated hearts appeared to reverse this trend (*p* = 0.074 and *p* < 0.05, respectively), as these treatments increased mitochondrial NAD(P)H in response to a higher workload. Interestingly, the Combined treatment did not recapitulate the individual results of SS‐31 and NMN and instead showed mitochondrial NAD(P)H levels that were unchanged when dobutamine was applied. Other metabolites did not show a significant difference between groups with increased workload, and these results are presented in Figure S8.

## 3 DISCUSSION

### 3.1 SS‐31 and NMN improve different aspects of heart health

SS‐31 and NMN treatments distinctly improve diastolic and higher workload systolic function, respectively. Since giving the drugs in combination conferred both diastolic and systolic improvements, but neither prevented nor further added to the individual improvements, the data imply that the drugs use two independent mechanisms to achieve these functional benefits. Furthermore, since the drugs only showed an effect in Old hearts, this appears to be a true rejuvenation of heart function in both cases.

In this cohort of mice, we found that NMN and the Combined treatment were effective at reducing age‐related hypertrophy of the heart while SS‐31 alone was not. However, in other cohorts of mice we have previously observed a modest but significant reduction in hypertrophy following SS‐31 treatment (Chiao et al., 2020). Given the small difference in magnitude between these new results and those already published, we believe this difference is primarily based upon statistical variation and not the underlying biology.

NMN appears to have a much more acute effect on the heart than SS‐31. While NMN achieves its peak functional effect after just 10 days of treatment, we have previously observed that SS‐31 treatment takes approximately 8 weeks to reach its peak effect on the heart (Chiao et al., 2020). NMN's effect was halved by 6 days after treatment cessation and completely gone after 10 days, whereas a partial SS‐31 effect persisted for several weeks after cessation of treatment (Chiao et al., 2020). These differing timelines fit with the hypothesis that SS‐31 carries out its effects by repairing mitochondria efficiency and reducing oxidative stress, followed by myocardial remodeling (Chiao et al., 2020), whereas NMN primarily works by boosting NAD(H) biosynthesis and thus enhancing myocardial energetic supply via oxidative phosphorylation.

Consistent with these proposed mechanisms, we did not detect any difference in mitochondrial coupling/efficiency in the Seahorse assay with unstimulated cardiomyocytes isolated from NMN‐treated hearts. This is in clear contrast to SS‐31, which improved proton leak and respiratory control ratio in the same assay (Zhang et al., 2020). We believe this is consistent with mitochondrial remodeling by SS‐31 that does not occur following NMN treatment. However, this does not mean that NMN does not have any effect on the mitochondria, as discussed below.

### 3.2 Metabolic crosstalk between SS‐31 and NMN and the fate of NAD^+^

The observation that NAM and MNA concentrations increased after NMN treatment is unsurprising, given that both are steps along the breakdown pathway of NAD^+^ (Bender, 2003). Thus, the increase in both metabolites is an expected result of increased NAD^+^ biosynthesis and greater turnover of NAD^+^ from its use as an enzyme cofactor, primarily for sirtuin deacetylases and poly ADP‐ribose polymerases (PARPs) (Pillai, Isbatan, Imai, & Gupta, 2005). This enhanced turnover of NAD^+^ may explain why NMN treatment does not significantly increase resting NAD(H) levels, as noted here and in previous study (Mills et al., 2016). However, it is less clear why NMN and SS‐31 given in combination did significantly increase the NAD(H) pool when neither drug alone did. This would appear to indicate a synergistic effect of the drugs. One plausible explanation is that SS‐31's demonstrated reduction of oxidative stress (Chiao et al., 2020; Hou et al., 2016) results in less PARP activation and therefore less NAD^+^ breakdown, allowing it to accumulate to higher levels in the cell.

Since NNMT, the enzyme that converts NAM to MNA and removes it from reentering the NAD^+^ salvage pathway of synthesis (Bockwoldt et al., 2019), was at undetectable levels in the heart, if present at all, conversion to MNA is likely occurring elsewhere in the mice, with the MNA found in the heart having entered from the circulation. One concern with increased MNA synthesis is the potential for depletion of methyl donors in the cell (Pissios, 2017). Neither the NMN or Combined treatment group showed a significant decrease in S‐adenosyl methionine (Appendix S1), the major methyl donor of the cell and the one utilized for MNA synthesis, so it does not appear that methyl donors were depleted to a significant level. However, enhanced usage of S‐adenosyl methionine likely does explain why the slight increase in choline induced by SS‐31 treatment was prevented by simultaneous treatment with NMN, since choline is used to produce S‐adenosyl methionine (Zeisel & Corbin, 2012).

Why SS‐31 treatment causes an increase in xanthine and choline, and why the addition of NMN prevents the accumulation of xanthine, is less clear. In the case of choline, the SS‐31 effect appears to be a restoration of an age‐related loss in the metabolite and could be due to changes in phospholipid production induced by SS‐31's remodeling of the mitochondrial inner membrane. Conversely, the increase in xanthine is a further movement away from the Young state and the reason for this change is unclear. Adenine is one source of xanthine, by its deamination to hypoxanthine (Harrison, 2002), and more adenine is expected to be used for NAD^+^ synthesis with the addition of NMN, providing one potential explanation for the NMN treatment preventing the SS‐31‐induced increase in xanthine. Overall, the differences in xanthine and choline appear to be minor metabolic consequences of the drugs but seemingly unrelated to the functional improvements in the heart.

Although a wide selection of metabolites was screened, there are more that could be assessed to provide valuable information on the aging heart and the effect of these drugs. Lipid metabolism is an area we believe demands further investigation in future studies with this model system. Measurement of metabolic enzyme levels and activity could also provide valuable information on the mechanisms of age‐related dysfunction and the restorative properties of these drugs.

### 3.3 SS‐31 and NMN restore PCr/ATP dynamics in the heart

In this study, we applied in vivo MRS to demonstrate that there is an age‐related shift in the dynamics of PCr/ATP in response to increased cardiac workload. Old Control hearts showed a drop in PCr/ATP with a higher workload, whereas this parameter remained stable in Young hearts. From this, we can conclude that mitochondria in Old hearts are less able to meet the energetic demand of a high work state and PCr decreases as a consequence of its high‐energy phosphates being utilized to maintain ATP status in myofibrils faster than oxidative phosphorylation can restore it. The attenuation of the effect of age on the PCr/ATP dynamics by SS‐31 and NMN treatment, both individually and in combination, suggests that both drugs improve the ability of cardiac mitochondria to meet the metabolic demand of the increased workload.

### 3.4 Proposed model of dynamic mitochondrial metabolism changes

While limited by signal to noise and high variance, the novel MRS method that we describe here presents an exciting new means to analyze metabolic changes in response to a stimulus in live mice. We believe that the mitochondrial NAD(P)H peak we have described consists primarily of NADH based on its dynamic behavior here and in our prior study in which it was first described (Conley et al., 2016), although we are unable to definitively say to what degree NADPH contributes to the peak. Taking all of the above results into consideration, we propose the following model to contextualize the differences in metabolic shifts seen between the mouse groups with the increase in cardiac workload.

Young hearts have a sufficient NAD(H) pool and undamaged mitochondria, which provide them with adequate ATP production capacity to meet a higher workload. As a result, NAD^+^ and PCr do not show any significant shifts from increasing workload, while mitochondrial NAD(P)H trends toward a slight decrease due to greater utilization. Old Control hearts have a diminished NAD(H) pool, so higher workload places a greater strain on the mitochondrial ATP production capacity, as evidenced by accumulating NAD^+^, decreasing PCr, and reduced high work systolic function.

SS‐31 in Old hearts appeared to improve the efficiency of ATP production in the mitochondria, as it normalized the shifts in PCr and NAD^+^ that resulted from increasing the cardiac workload. A reason for this might be that mitochondrial NAD(P)H showed a reverse in the direction of change with increasing workload following SS‐31 treatment, indicating that the supply‐demand matching of NADH production was improved, with supply now outpacing demand rather than lagging behind. This implies a potential improvement in the efficiency of the tricarboxylic acid (TCA) cycle. Although several TCA cycle intermediates were evaluated by the general metabolomics screen, which did not show differences resulting from SS‐31 treatment (Appendix S1), this does not rule out the involvement of the TCA cycle since these measurements were only collected from tissue taken at rest. We have also recently demonstrated that there is a direct interaction between SS‐31 and TCA cycle enzymes involved in alpha‐ketoglutarate metabolism (Chavez et al., 2019). Other reasons why ATP production may be enhanced by SS‐31 lie in its direct effects on the mitochondrial inner membrane and inner membrane proteins. We have recently demonstrated that SS‐31 is highly effective at preventing an age‐associated excess protein leak across the mitochondrial inner membrane, which appears to be mediated by the adenine nucleotide translocase (Zhang et al., 2020). Reduced production of reactive oxygen species, which otherwise cause damage to mitochondrial proteins and consume NADPH through antioxidant regeneration, also occurs and is another likely mechanism for the improved efficiency (Chiao et al., 2020). However, these improvements are apparently not sufficient for SS‐31 to restore higher work systolic function.

NMN also normalized PCr levels under the higher workload in Old hearts, but by a seemingly different mechanism than SS‐31. NMN‐treated hearts were the only group to show a simultaneous increase in both NAD^+^ and mitochondrial NAD(P)H content. This could indicate that NMN‐treated hearts responded to increased workload by rapidly increasing the synthesis of NAD^+^ in order to raise mitochondrial NAD(H) levels and provide a higher capacity for work in situ. This seemingly improved capacity allowed these hearts to match the Young state in high work systolic function.

The Combined treatment in Old hearts appeared to best recapitulate the Young state. This group did not show any significant shifts in PCr, NAD^+^, or mitochondrial NAD(P)H following the increase in workload, which we believe to be due to the higher energetic capacity afforded by the restoration of resting NAD(H) levels. These hearts matched the high work systolic function of the Young state, while also exhibiting improved diastolic function. Thus, the combination of SS‐31 and NMN, which together appear to increase fuel supply and coupling efficiency while limiting excess oxidative stress, provides a synergistic improvement in energetic capacity so that supply can fully meet demand.

### 3.5 Mechanisms of diastolic improvement by SS‐31

It is less clear whether the metabolic changes described above are directly responsible for the improvement in diastolic function resulting from SS‐31 treatment. Metabolic changes certainly do play a role in diastolic dysfunction, as it is well established that ADP levels have a regulatory role in sarcomere stiffness with higher ADP inhibiting relaxation and increasing diastolic stiffness (Sequeira et al., 2015). The previously reported effects of age and SS‐31 on mitochondrial coupling in cardiomyocytes (Zhang et al., 2020) are consistent with a role for altered ADP levels in the changes in diastolic function reported here. However, we did not detect any significant changes in ATP or ADP at rest in Old hearts treated with SS‐31 in our metabolomics analysis (Appendix S1). Furthermore, we found by MRS that PCr/ATP at rest was unaltered by age or SS‐31 (Figure S10), also indicating a lack of difference in resting ADP levels. In addition, the effects on SS‐31 mitochondrial coupling we have reported appear to be rapid onset, while the improvements in diastolic dysfunction require longer term treatment. An alternative mechanism, for which we have previously published direct evidence (Chiao et al., 2020), is the effect of SS‐31 on post‐translational modification of myofibrillar contractile proteins. In particular, SS‐31 greatly reduced age‐related increases in the oxidation state of the proteome, including contractile proteins, and restored the phosphorylation levels of the sarcomeric protein myosin‐binding protein C, cardiac‐type (cMyBP‐C) to the young state. These changes are consistent with improved relaxation of the cardiac sarcomere, a critical aspect of diastolic function (Gilda & Gomes, 2017; McNamara, Singh, & Sadayappan, 2019).

### 3.6 Conclusions

By testing SS‐31 and NMN together in mice, we were able to provide new insight into the effects and mechanisms of each drug in the heart. Excitingly, this work shows that NMN and SS‐31 can be given in combination to rejuvenate both diastolic and systolic aspects of ventricular function, boost NAD(H) content better than NMN given alone, and stabilize mitochondrial NAD(H) dynamics. These results may have strong clinical relevance and imply that synergistic treatment with the two drugs in combination may be more effective at treating age‐related heart dysfunction than either given alone. This is also an illustration that complimentary use of appropriate drug combinations may become a more general model for achieving optimal healthspan extension.

## 4 EXPERIMENTAL PROCEDURES

### 4.1 Animal use and care

All mice used in this study were males of the C57BL/6 strain. Young and Old mice were obtained from the National Institute on Aging Charles River colony and further aged to 5–6 and 24 months, respectively, before starting the study. Mice were kept under diurnal conditions with ad libitum food and water. Mice were housed at 20°C under diurnal conditions in an AAALAC‐accredited facility under Institutional Animal Care and Use Committee supervision with ad libitum access to food and water. Old mice were randomly assigned to Control, SS‐31, NMN, or Combined treatment groups.

### 4.2 Drug administration and treatment groups

SS‐31 was provided by Stealth BioTherapeutics (Newton, MA) and administered at a 3 mg/kg body weight/day dosage through osmotic minipumps (ALZET, Cupertino, CA) implanted surgically under the skin on the left dorsal side of the mice. Pumps were modified to replace the metal stem with PEEK tubing, according to the manufacturer's instructions, for compatibility with magnetic resonance spectroscopy. After 4 weeks, the original minipump was surgically removed and a new minipump was implanted to continue the SS‐31 administration for another 4 weeks.

NMN was obtained from the Imai laboratory (Washington University in St. Louis, MO) and administered through ad libitum drinking water with a concentration based on each cage's measured water consumption rate and mean mouse body weight to approximate a 300 mg/kg body weight/day dose. NMN water was the only source of hydration given to these mice and was replaced every 3–7 days based upon prior tests showing that it remains stable in water at room temperature for at least 1 week (Mills et al., 2016).

Combined treatment mice received SS‐31 through minipumps while simultaneously receiving NMN water as their exclusive source of hydration as described above.

Initially, two different controls groups were used: mice with osmotic minipumps containing only saline as a control for SS‐31 and mice without minipumps receiving standard water as a control for NMN. However, an analysis of functional and MRS results revealed that there were no significant differences between these two control groups (Figure S9). Thus, the two control groups were integrated into a single control for all treatment conditions.

### 4.3 Echocardiography

Mice were anesthetized by 0.75%–2% isoflurane, and echocardiography was performed using a Siemens Acuson CV‐70 (Munich, Germany) equipped with a 13 MHz probe. Heat support and heart rate monitoring were provided throughout the procedure with mouse heart rates being maintained in the range of 450–550 bpm at the low work state. Analysis of systolic function included an injection of 3 µg/g body weight dobutamine in order to induce a higher cardiac workload and analyze maximal function. High work echocardiography was performed once the heart rate increased at least 100 bpm and remained stable.

### 4.4 Euthanasia and tissue handling

Mice were euthanized by live cervical dislocation. Hearts were immediately removed, flushed with PBS to remove blood, and weighed. A 2‐mm section was removed from the ventricles for histology, and the remaining tissue was cut into ~2 mm^3^ chunks and snap‐frozen in liquid N_2_ to store for further processing. Frozen tissue was mechanically lysed into a fine powder using a Tissuelyser II (Qiagen) prior to preparation for LC‐MS/MS‐based measurements.

### 4.5 Targeted NAD^+^ metabolomics

Levels of NAD^+^, NADH, NADP^+^, NADPH, NA, NAAD, NAM, NR, and NMN at treatment endpoints were measured by Ultra Performance Liquid Chromatography coupled with Mass Spectrometry as previously described (Trammell & Brenner, 2013), with some modifications. Under CO_2_ vapor, lysed tissue was dissolved in chilled buffered methanol solution (75% methanol, 25% 10 mM HEPES pH 7.8) spiked with stable isotope‐labeled internal standards of NAD^+^ and NADH for normalization, and brought to a final concentration of 250 mg/ml. Samples were then spun down repeatedly at 16,000 *g* for 10 min and transferred to new tubes until no pellet formed. Pellets were saved for protein quantification as an additional normalization step. Supernatants were filtered using 4‐mm and 0.22‐µM syringe filters (MilliporeSigma, Burlington, MA). 5 µl of the extract was separated on a BEHAmide column (Waters, Milford MA) using an Acquity UPLC (Waters) and analyzed with a Xevo TQ (Waters) in multiple reaction monitoring mode (MRM). LC solvents were A: H_2_O with 10 mM ammonium acetate and 0.4% NH_4_OH; and B: 95:5 acetonitrile H_2_O with 10 mM ammonium acetate and 0.4% NH_4_OH for all metabolites. Samples were run alongside an external standard curve for quantification.

### 4.6 General targeted metabolomics

Heart tissue samples were homogenized in water and methanol with spiked stable isotopes as internal QC standards. Samples were purified by pelleting proteins, drying of the solution via Speedvac (Thermo Fisher Scientific), and reconstituting in HILIC solvent. Protein pellets were analyzed by BCA (Thermo Fisher Scientific) as a normalization step. LC‐MS/MS‐based metabolomics targeting a list of 369 metabolites was performed on a system consisting of Shimadzu Nexera XR LC‐20AD pumps (Kyoto, Japan) coupled to a Sciex (Framingham, MA) 6500+ triple quadrupole spectrometer operating in scheduled MRM detection mode through the Sciex Analyst 1.6.3 software, as described elsewhere (Nagana Gowda, Djukovic, Bettcher, Gu, & Raftery, 2018). The system includes a dual‐column setup with dedicated columns for positive ionization mode and negative ionization mode. Metabolite concentrations were quantified using Sciex MultiQuant 3.0.2 software.

Analysis of the dataset was performed using R (version 3.6.0). In order to remove the systematic variation between samples, we performed a Cyclic LOESS normalization, which had good performance using a MS benchmark dataset (Li et al., 2017). This normalization step is implemented using the limma R package (Ritchie et al., 2015). Missing values are known to be a problematic issue for mass spectrometry‐based data. Previous studies have summarized the three missing value mechanisms in terms of MS‐based data (Lazar, Gatto, Ferro, Bruley, & Burger, 2016; Wei et al., 2018): Missing Completely At Random (MCAR), which corresponds to the combination and propagation of multiple minor errors or stochastic fluctuations. Each missing value cannot be directly explained by the nature of the feature or by its measured intensity. Missing At Random (MAR) assumes the possibility of a variable being missing is determined by other observed variables, for example, inaccurate peak detection. Missing Not At Random (MNAR) corresponds to censored missing values caused by compound abundances that are below the limits of quantification (LOQ), a.k.a. left‐censored data. For the metabolomics data, the missing value mechanism is believed to be MNAR. All the metabolites with >40% missingness were excluded, and a total of 191 metabolites were included in the imputation step (roughly 0.9% of the data is missing values after filtering). We used a quantile regression approach for the imputation of left‐censored missing data (QRILC), which has been suggested as the favored imputation method for left‐censored MNAR data (Wei et al., 2018). Briefly, QRILC performs imputation by replacing missing values with random draws from a truncated distribution with parameters estimated using quantile regression. This was implemented using the imputeLCMD R package. Imputation was performed after the cyclic LOESS normalization. We fit a linear model to the normalized and imputed metabolomic data using the Bioconductor limma package while adjusting the preparation batch and protein concentration as covariates in our model. The limma package uses empirical Bayes moderated statistics, which improves power by ‘borrowing strength’ between metabolites in order to moderate the residual variance (Smyth, 2004). Sample size for each group after removing outliers was Young = 12, Old Control = 10, Old NMN = 11, Old SS‐31 = 12, Old Combined = 8. False discovery rate (FDR) was limited to 10% using the Benjamini–Hochberg method.

Heatmap generation was performed in R using the “heatmap” function to create a row‐scaled image based upon the top 20 significantly altered canonical pathways in the Young vs. Old Control comparison as determined by IPA (Qiagen).

### 4.7 ^31^P‐magnetic resonance spectroscopy

Magnetic resonance spectroscopy was performed on live mice using a 4.7 Tesla instrument operated by the Translational Center for Metabolic Imaging at the University of Washington. Mice were placed into a custom apparatus with coils for both ^1^H and ^31^P NMR and anesthetized minimally via nose cone with 0.75%–1.5% isoflurane. Heart rate, respiratory rate, and temperature were monitored using a Model 1030 Monitoring & Gating System (Small Animal Instruments, Inc., Stony Brook, NY). Anesthesia was adjusted as needed to maintain respiratory rate at 100–130 bpm. Temperature was also adjusted as needed to keep mice at a body temperature of 37°C. Mice were equipped with an intraperitoneal catheter connected to a line of dobutamine to allow for remote injection without altering the position of the mouse.

Triggered, multi‐slice, gradient echo, ^1^H‐magnetic resonance images were acquired (TR = 160 ms, TE = 4 ms, with a 2 mm slice thickness) to place a voxel isolating the heart for ^31^P ISIS (Ordidge, Connelly, & Lohman, 1986). ^31^P ISIS spectra (TR = 3 s) were the result of 96 averages (acquiring a total of 768 acquisitions) using 2 ms hyperbolic secant 180^o^ pulses during localization, but a nominal 90° square (50 μs), non‐selective pulse for readout. Typical voxel size was ~0.8 cm^3^ to sample the entire heart. Data were collected over a 38‐min acquisition with gating based upon heart rate and respiratory rate to ensure the position of the heart was constant across measurements. Following the first acquisition, mice were injected with 5 µg/g dobutamine. The second acquisition was started once the elevated heart rate became stable.

Manual phase adjustment of the spectra, alignment of PCr to −2.54 ppm, and quantification of PCr and γ‐ATP integrals were performed in TopSpin (Bruker, Billerica, MA). Baseline correction of spectra was performed using Mnova software (MestreLab Research, S.L., Santiago de Compostela, Spain).

OriginPro software (OriginLab Corporation) was used to model the fitting of α‐ATP, NAD^+^, cytosolic NADH, NADP^+^, cytosolic NADPH, UDP‐glucose, and mitochondrial NAD(P)H peaks to the baseline‐corrected experimental spectra for quantification of these molecules. Lorentzian peaks were positioned and modeled as singlets, doublets, or quartets based upon analysis chemical standards on the instrument under physiological (12 mM Mg2+, pH 7.4) conditions (Figure S10). Peak width was variable across different experimental spectra but was kept consistent for NAD^+^, cytosolic NADH, NADP^+^, cytosolic NADPH, and UDP‐glucose within each fit. Mitochondrial NAD(P)H peak width was allowed to vary within 1.5‐ to threefold that of the other peaks. Best fits were generated automatically based on this model, with no manual adjustment used. Peaks were integrated automatically, and non‐ATP metabolites were normalized to α‐ATP for relative quantification. OriginPro macros are available upon request.

### 4.8 Statistical analysis

Statistical analysis of large‐scale metabolomic data is described in the above section. Other statistical analyses were performed using Prism (GraphPad Software, San Diego, CA). One‐way ANOVAs were used for measurements with a single time point or state per group, and two‐way ANOVAs were used to compare measurements with multiple time points or states for each group. One‐sample t tests were used to determine whether delta means were significantly different from 0. All results are plotted as means ± SEM.

## CONFLICT OF INTEREST

The authors declare that they have no conflict of interest.

## Supporting information

Figure S1‐S10

Appendix S1

## Data Availability

Supplemental methods and results can be found in the Supplemental Data document. The full general targeted metabolomics dataset can be found in the Appendix S1. In accordance with *Aging Cell's* data availability policy, these data are also available at the National Metabolomics Data Repository (NMDR) (https://www.metabolomicsworkbench.org), which is supported by NIH grant U2C‐DK119886. It has been assigned Project ID PR000908 and can be accessed directly via its Project https://doi.org/10.21228/M8B11Q.
