# Supplementary material for: SS‐31 and NMN: Two paths to improve metabolism and function in aged hearts
Source: Aging Cell. 2020 Aug 11;19(10):e13213. doi: 10.1111/acel.13213 (PMC7576234; doi:10.1111/acel.13213)
Supplement: Supplementary file 1 — Figure S1‐S10 [file ACEL-19-e13213-s001.docx]

**SUPPLEMENTAL EXPERIMENTAL PROCEDURES**

**Cardiomyocyte Isolation and Seahorse Assay**

Ventricular cardiomyocytes were isolated from mouse hearts following the same protocol published previously (Chiao et al. 2020). Briefly, animals were euthanized by live cervical dislocation and hearts were immediately removed from the chest and perfused with oxygenated Tyrode’s solution containing 300U/ml collagenase II and 0.5mg/ml hyaluronidase at 37°C. Dissociated cells were counted and plated in 24-well plates with 1 mM pyruvate and 5 mM glucose as the fuel sources for Seahorse XF24e Extracellular Flux Analyzer analysis (Agilent, Santa Clara, CA) with subsequent additions of oligomycin, FCCP and rotenone + antimycin A.

**Western Blotting**

Proteins were extracted from frozen heart tissues with RIPA buffer with HALT protease inhibitor and quantified by BCA protein assay. Equal amount of proteins (15 µg) were resolved on a 4-12% NuPAGE Bis-Tris gel and transferred to PVDF membrane. A Pierce Reversible Protein Stain Kit was used to detect total proteins for loading control. All listed materials were obtained from Thermo Fisher Scientific (Waltham, MA).

The primary antibody used in immunoblotting was NNMT #24912 (Cell Signaling Technology, Danver, MA). The secondary antibody used was donkey anti-rabbit IgG secondary antibody (Thermo Scientific). AlphaView Software (Protein Simple, San Jose, CA), was used for image acquisition and quantification.

**SUPPLEMENTAL FIGURES**

**Supplemental Figure 1. Dobutamine-induced heart rate changes in mice.** Heart rates were recorded from monitoring during MRS acquisitions. N = 11 Young, 20 Old Control, 14 Old SS-31, 17 Old NMN, 12 Old Combined.

**Supplemental Figure 2. Assessment of low work percent fractional shortening by echocardiography**. Endpoint = 8 weeks of treatment. N = 17 Young, 29 Old Control, 19 Old SS-31, 18 Old NMN, 10 Old Combined.

**Supplemental Figure 3. Assessment of SS-31 longitudinal effect on Young mouse heart diastolic function by echocardiography.** N = 4.

FCCP

Oligomycin

**Supplemental Figure 4. Seahorse comparison of Old Control and NMN isolated cardiomyocyte mitochondrial function.** Vertical lines indicate timing of material injections. N = 4 NMN, 2 Control.

**30 kDa**

**Mouse Liver**

**A549 Positive Control**

**HELA Positive Control**

**Young Mouse Heart**

**Old Control Mouse Heart**

**Old NMN Mouse Heart**

**Old Combined Mouse Heart**

**Old SS-31 Mouse Heart**

**
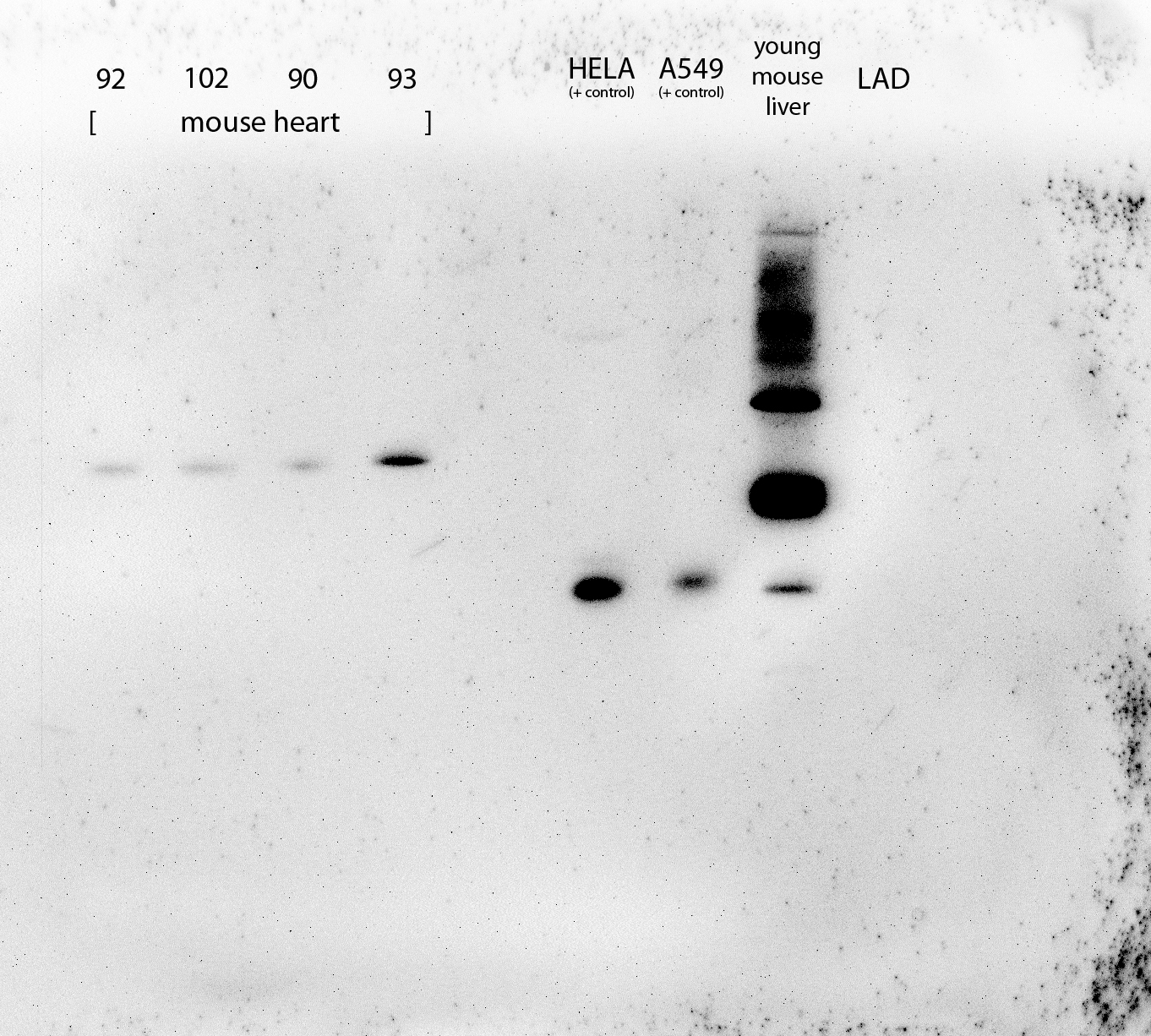
**

**Supplemental Figure 5. Western blot of NNMT.** NNMT was detectable at ~28 kDa in mouse liver and positive controls but not in any mouse heart samples.

**
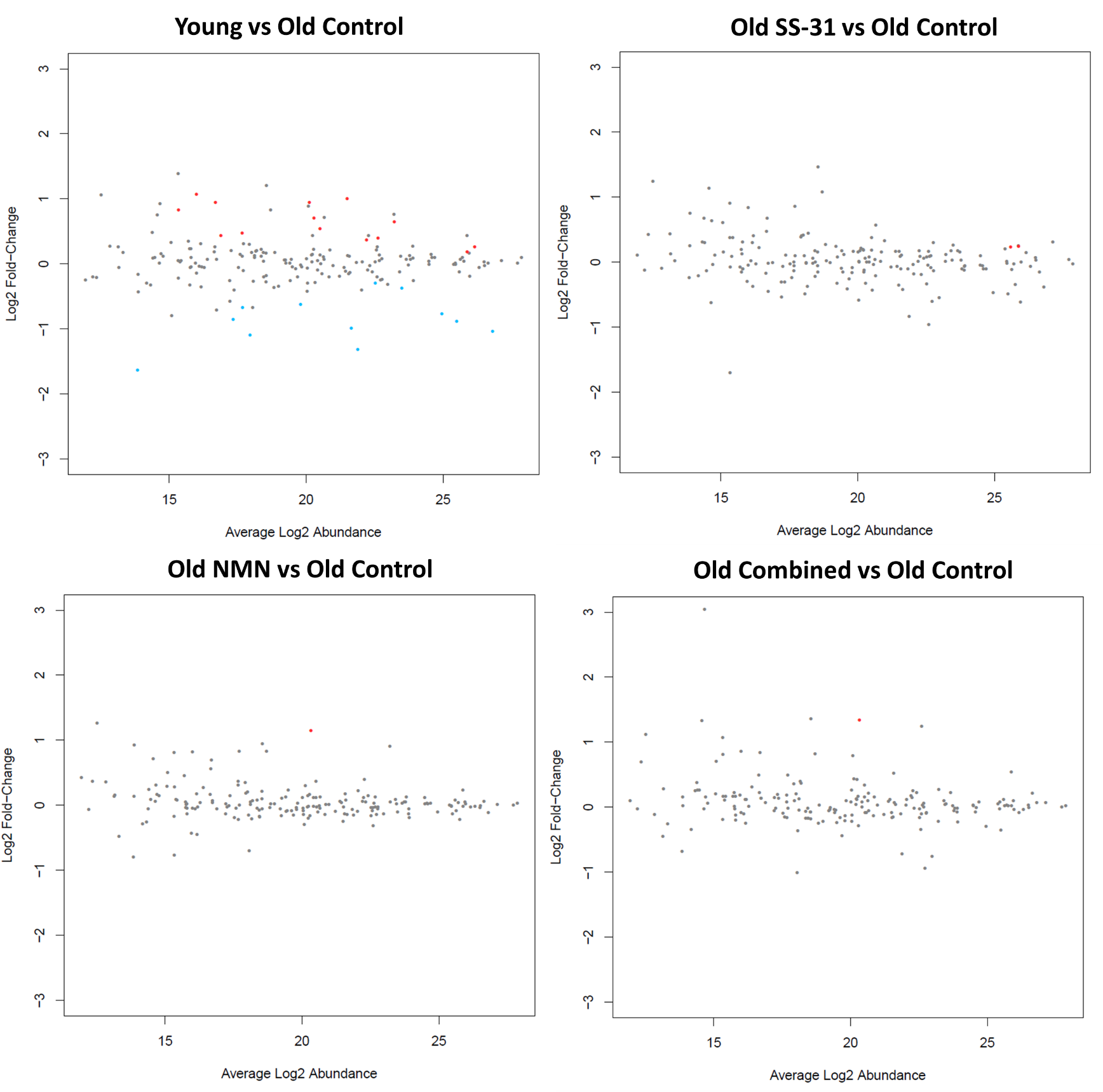
**

**Supplemental Figure 6. Mean difference plots of metabolomic data.** Red = significantly increased (FDR<0.1). Blue = significantly decreased (FDR<0.1). Gray = no significant difference.

**Supplemental Figure 7. ^31^P Magnetic Resonance Spectroscopy Work Jump Measurements of Metabolites.** Measurements of (A) NADH, (B) NADP^+^, and (C) UDP-glucose in mouse hearts derived from OriginPro model. N = 11 Young, 10 Old Control, 9 Old SS-31, 11 Old NMN, 10 Old Combined.

**Supplemental Figure 8. Comparison of Functional and Bioenergetic Measures in Saline and Untreated Old Controls.** No comparisons reach significance by ANOVA or T test. (A and B) N = 9 Saline, 20 Untreated. (C) N = 18 Saline, 22 Untreated. (D) N = 8 Saline, 12 Untreated. (E) N = 5 Saline, 6 Untreated. (F) N = 5 Saline, 5 Untreated.

**
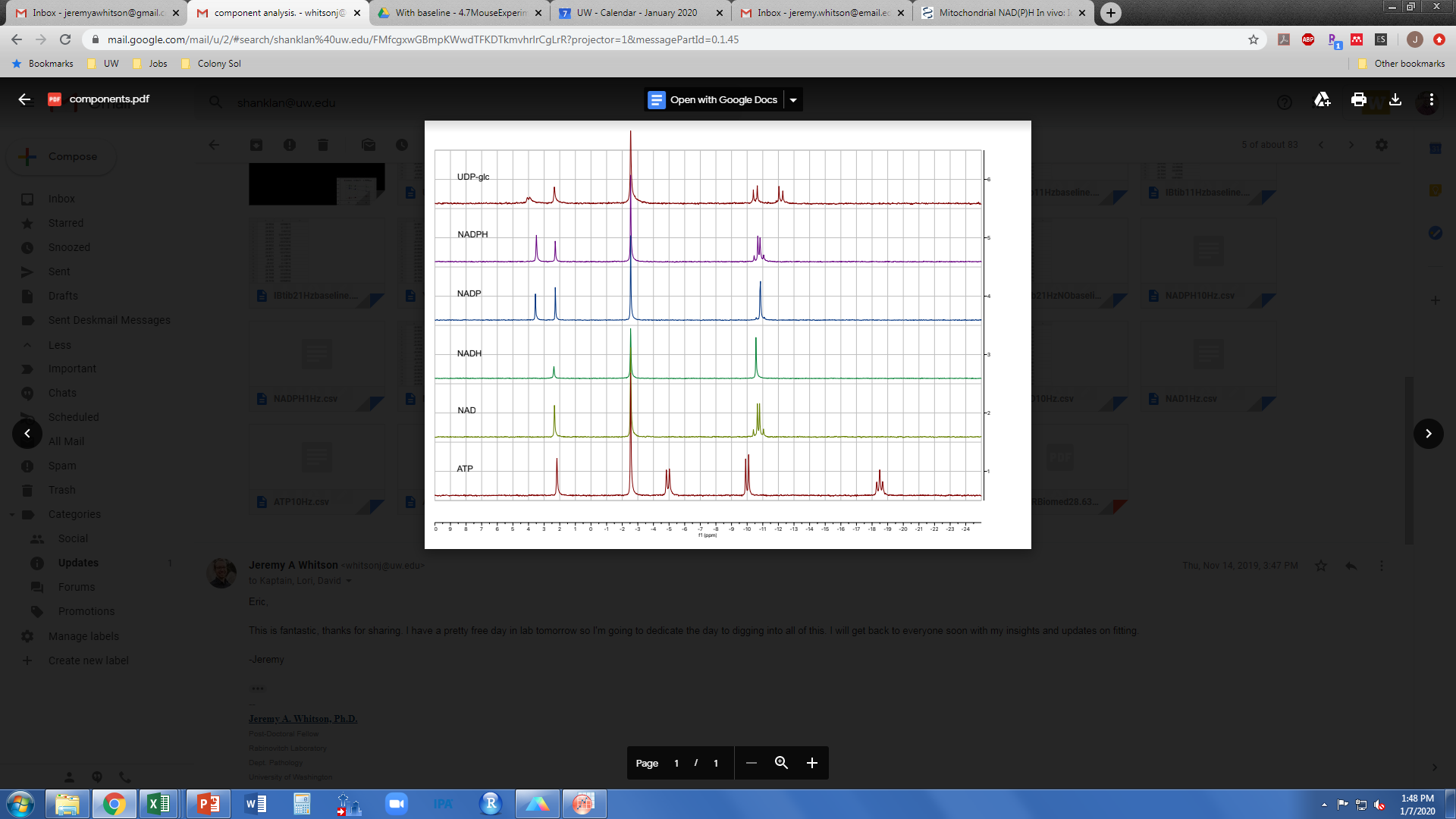
**

**Supplemental Figure 9. ^31^P spectra of chemical standards.** All spectra are adjusted to PCr at -2.54 ppm and broadened to 1 Hz.

**Supplemental Figure 10. Resting PCr/γ-ATP of mouse hearts derived from ^31^P-MRS.** N = 11 Young, 17 Old Control, 13 Old SS-31, 17 Old NMN, 13 Old Combined.

**WORKS CITED**

Chiao, Ying Ann et al. 2020. “Late-Life Restoration of Mitochondrial Function Reverses Cardiac Dysfunction in Old Mice.” *bioRxiv*: 2020.01.02.893008.
